# Investigating the possibility of extending the BWR cycle length for 15 years of operation by mixing highly enriched UO_2_ fuel with burnable absorbers

**DOI:** 10.1038/s41598-023-37615-x

**Published:** 2023-06-29

**Authors:** Sayed Saeed Mustafa

**Affiliations:** Faculty of Science, Zagazig University, Zagazig, Egypt

**Keywords:** Nuclear physics, Theoretical nuclear physics

## Abstract

This paper investigates the possibility to extend the cycle length of boiling water reactor bundles to 15 years of operation with three different burnable poisons; gadolinium, erbium, and boron carbide. This can be carried out by mixing highly enriched UO_2_ fuel (15–19.9% U-235) with high concentrations of Gadolinium oxide (3–14% Gd_2_O_3_) or Erbium oxide (2–4% Er_2_O_3_).The boron carbide B_4_C was modeled as (Al_2_O_3_-B_4_C) rods in the bundle guide tubes. MCNPX code 2.7 was used to evaluate infinite multiplication factor (K-inf), power distribution, peaking factor, void reactivity coefficient, fuel cycle length, depletion of U-235, and fissile inventory ratio for the three designs at 40% void. The MCNPX simulation showed that introducing gadolinium rods at the bundle periphery has the advantage of lowering reactivity swing throughout the exposure range. The uniform distribution of erbium in all fuel rods contributed to the flattening of peaking factor at all the burnup stages. For the B_4_C design, the author found that the assembly with B_4_C–Al performs best in terms of reactivity flattening when five of the B_4_C–AL_2_O_3_ rods are positioned in the central region of the assembly. Furthermore, the fuel temperature coefficient is more negative for gadolinium design at all burnup stages. On the other hand, the boron model delivers the lowest control rod worth. Finally, the moderator temperature coefficient is more negative for erbium and WABA designs due to the enhanced thermal neutrons capture by the effect of the strategic arrangement of WABA rods and the uniform distribution of erbium.

## Introduction

As demonstrated by the projects conducted by the Nuclear Energy Research Initiative (NERI) programme, the high fuel burnup is a significant issue in promoting innovative reactor concepts. The high fuel burnup concept allows for a higher fuel utilization and extracting more power from this fuel^1,2^. Light water reactor fuel cycles with high fuel burn-up (over 100 GWd/ton) typically require large amounts of fissionable materials that can sustain the fission chain reaction for a long time^3^.

The long cycle lengths for light water reactors (PWR and BWR) can be achieved by two main methods. The first method is to maximize the current regulation limit for the fuel discharge burnup. In the current reactor designs, the fuel exposure limit is set at 60 or 70 GWd/ton for fuel enrichment of 5–6% U-235^4^. Increasing this limit to proposed higher burnup values contributes to better fuel utilization and minimizing the amount of fuel that should be stored in spent fuel pools and in dry storage^5^. It is expected that the increased burnup limit has the advantage of reducing or eliminating the amount of refueling, which in turn improves the capacity factor. The second method is to increase the fuel enrichment percentage. The limit of enrichment in most current light water reactors is 5% U-235. Increasing this limit to a proposed 20% U-235 can successfully improve the fuel cycle economics^6^.

A number of technical obstacles must be taken into consideration to make extending the cycle length concept feasible. The loss of coolant accident and the reactivity insertion accident are two of the major obstacles that must be overcome for prolonging the fuel cycle length. As known, the integrity of nuclear fuel is reduced when it remains for a long period in the reactor core. This leads to the crack of fuel pellets and the release of enormous amounts of fission product gases. This contributes to an increase in the plenum pressure that causes stress on the cladding material. Furthermore, there is an increase in the corrosion and hydrogen absorption in the cladding material. These issues affect the integrity of cladding and make it easy to crack. During the normal operation of the reactor, these obstacles do not threaten the cladding integrity. Nevertheless, some undesirable events such as fuel fragmentation and fuel pin bursting may occur at 62 GWd/ton. Therefore, these obstacles must be overcome to increase the burnup limit over 62 GWd/ton^7^.

In Boiling Water Reactors, the production of a high conversion ratio is one of the effective methods that can satisfy a super long operating cycle (over 15 years). Such a high conversion ratio can be achieved either by the utilization of plutonium mixed oxide (MOX) or by decreasing the fuel to moderator ratio. Both of these approaches harden the neuron spectrum and enhance the fissile inventory ratio^8,9^. As stated before, the homogeneous mixing of uranium oxide fuels with burnable absorbers is also a nother method that can raise the conversion ratio. Gadolinium, erbium, and boron carbide are selected in this study because of their known neutronic characteristics in a typical Light Water Reactor environment^10^.

Enriched gadolinium (Gd-157) is a candidate absorber for the BWR long cycle operation because its implementation necessitates increasing the uranium enrichment to compensate for the reactivity penalty caused by the even-numbered gadolinium isotopes^11^. Many methodologies have been developed for improving the capacity factor, minimizing the local cost, and reducing the proliferation potential of very long BWR cores. These methodologies focused on controlling and maintaining criticality with control rods and burnable poisons and concluded that the very long cycle operation can be attained by increasing the fuel enrichment or adopting a high conversion tight pitch lattice. Increasing the fuel enrichment requires long-lived burnable poisons and control rods that suppress the initial excess reactivity^12–15^. The current study focuses only on the neutronics aspect of the long simplified boiling water reactor; as such, it does not cover mechanical, structural, thermodynamics and fluid properties of the fuel loaded in the core. The following points depict characteristics of the burnable absorbers used in the simulation.

### Gadolinium oxide

From the point of view of nuclear and reactor physics, gadolinium oxide (Gd_2_O_3_) is a strong burnable absorber with a high absorption cross-section. When it is distributed properly in the reactor core, it can minimize the reactivity swing over the core life. In this study, 14 gadolinium rods are distributed in the bundle at the periphery. Mixing gadolinium oxide with uranium oxide results in reducing the heavy metal content and shortening the core life cycle. Moreover, after the depletion of the main neutron-absorbing gadolinium isotopes (i.e., Gd-155 and Gd-157), a residual negative reactivity remains due to the presence of other gadolinium isotopes that are not destroyed^16,17^.

### Erbium oxide

Erbium-167 is one of the natural erbium isotopes that has a smaller thermal absorption cross section than that of Boron-10 and Gadolinium-157. In PWRs, erbium oxide (Er_2_O_3_) can be mixed homogeneously with uranium oxide fuel (UO_2_). The utilization of such a mixture causes a reduction in the core cycle length. The smaller absorption cross-section of erbium participates in a flattened power distribution owing to its slow consumption; this usually minimizes radial power peaking in the erbium oxide fueled cores^18^.

### Wet annular burnable absorber (WABA)

WABA is one of the burnable absorbers that can be modelled in the guide tubes that do not contain control rods. It is classified as a discrete burnable absorber with annular pellets of Al_2_O_3_–B_4_C (i.e., with 14 w/o B_4_C) and a wet water-filled central region. This absorber provides several benefits over other absorbers, such as increasing neutron moderation, reducing neutron absorption, and better absorber depletion. The discrete burnable absorber rods are modelled in the reactor core at temperatures below 900 K during normal operation^19,20^.

In the current research, the reactivity lifetime of light-simplified boiling water reactors is extended by mixing a highly enriched UO_2_ fuel, (15–19% U-235), with an enriched gadolinium that compensates for the high initial reactivity. The main objective of such a fuel design is to keep the K-infinity value around 1.05 over 15 years of operation. The neutronic and burnup calculations are carried out by the MCNPX code at the assembly (bundle) level. These calculations are conducted at 40% void, assuming that this is the average void at nominal values for the fuel and moderator temperatures. The calculations of the present work involve the following steps.

1. Modeling three BWR designs, (Gadolinium, Erbium, Boron Carbide), by MCNPX code
2. Comparison of the MCNPX code results with those of CASMO-4 code^21^.
3. Analyzing the effect of 40% and 80% voids on the reactivity, peaking factor, build up of Pu-239, thermal neutron fraction and fissile inventory ratio for the enriched gadolinium bundle.
4. Performing the depletion calculations for the enriched erbium bundle.
5. Performing the depletion calculations for the natural boron design
6. Comparison of the safety parameters as K-eff, power distribution, peaking factor, void reactivity coefficient, U-235 depletion, and thermal neutron fraction at 40% void to understand the inherit neutronic characteristics of each assembly with various types of burnable poison.

The novelty of this work is the proposed strategic placement of burnable poisons in the BWR designs. We can see the new arrangement of gadolinium at the periphery (next to water gap around the bundle) resulted in lowering the K-inf swing throughout the exposure range. On the other hand, the new arrangement of WABA rods in the natural boron bundle allows the variation of K-inf with burnup to be very small. This was conducted by positioning the WABA rods in the central region of the bundle. In this situation, the fuel rods located at the bundle periphery will deplete more slowly because they will be subjected to low thermal flux. After the depletion of Boron-10 in the central region, the peripheral fuel rods will start to burn. As a result, the K-inf behaviour from 30 to 100 GWd/ton is stabilized (flattened). This will increase the inherent safety of the reactor operation. Moreover, some previous works as reported in references^11,21^ are based on deterministic transport theory codes as CASMO-4 code. But, the present work was performed by a new methodology that depended on the probabilistic MCNPX code with the cross-section library ENDF/B-VII.1. Therefore, this work is considered to be the first to simulate the proposed BWR designs to extend their cycle length to 15 years using the MCNPX code.

## Conceptual BWR bundle design with burnable poison loading

The three reference design bundles under consideration were modelled to extend their cycle operation to 15 years. Increasing fuel enrichment, resizing the fuel pellet, and increasing the physical density of fuel are different methodologies that can be utilized for this purpose^22^.

**Figure 1:**
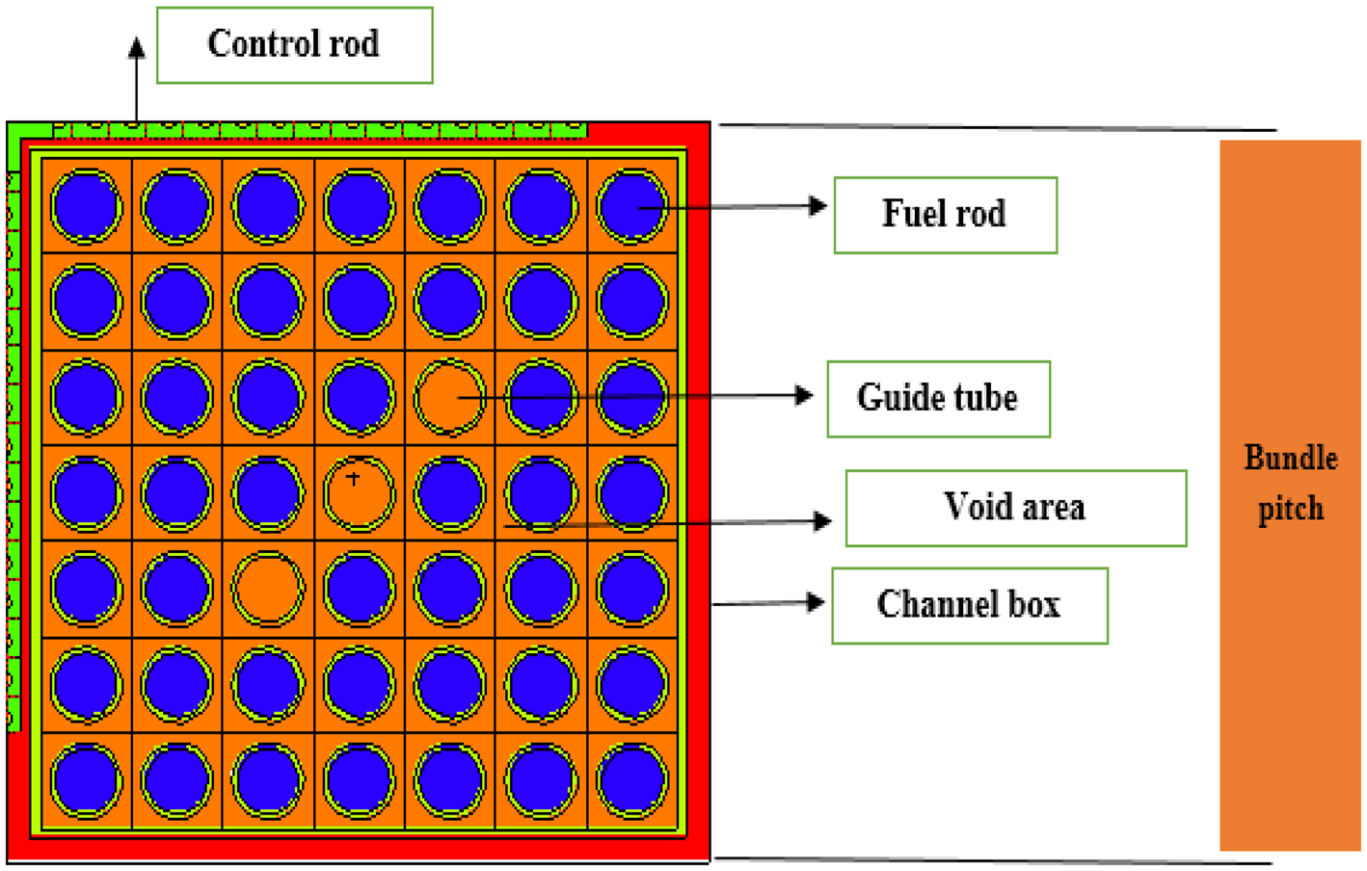
Fuel bundle horizontal view by MCNPX code.

The fuel bundle design specification is summarized in Fig. 1 and Table 1. The reference core design used in this study is a BWR design producing 900 MW of thermal power, which corresponds to 15 years. This core consists of 956 fuel assemblies. Each assembly consists of a 7 × 7 fuel rod array with a 2.0 m active length, and the bundle size is 0.7 times that of the current BWR bundle. The fuel bundle cell area is about half that of the typical BWR, and the moderator to fuel ratio is approximately 10% higher than the typical 8 × 8 BWR fuel design^23,24^.The specification of reference core design is provided in Table 2.

**Table 1:** Specification of BWR fuel bundle.

Parameter	Value
Active fuel length	200 cm
Bundle pitch	10.9 cm
Outer water gap width	1.3 cm
Channel box thickness	0.13 cm
Fuel rod array	7 × 7
Number of fuel rods	46
Number of water rods	3
Fuel rod pitch	1.26 cm
Fuel rod diameter	1 cm
Fuel pellet diameter	0.85 cm
Fuel pellet stuck density	95%TD (Theoretical density)
Gd-157 concentration in Gadolinium	80%
Control rod thickness	0.7 cm
Central steel region length	2 cm
Control rod absorber material	B_4_C
Control rod absorber radius	0.18 cm
Control rod absorber blade length	7.35
Control rod absorber tube pitch	0.5 cm
Control rod structural material	Stainless steel

**Table 2:** Specification of BWR core design.

Parameter	Value
Output thermal power	900 MW
Operating cycle length	15 years
Power density	40 kW/L
Bundle array	34 × 34
Number of control rods	225
Control rod insertion direction	Upper entry
Reactor dome pressure	7.2 Mpa
Feed water temperature	215 °C
Core flow rate	12,000 t/h
Cycle exposure	111 GWd/t

### Gadolinium reference design

**Figure 2:**
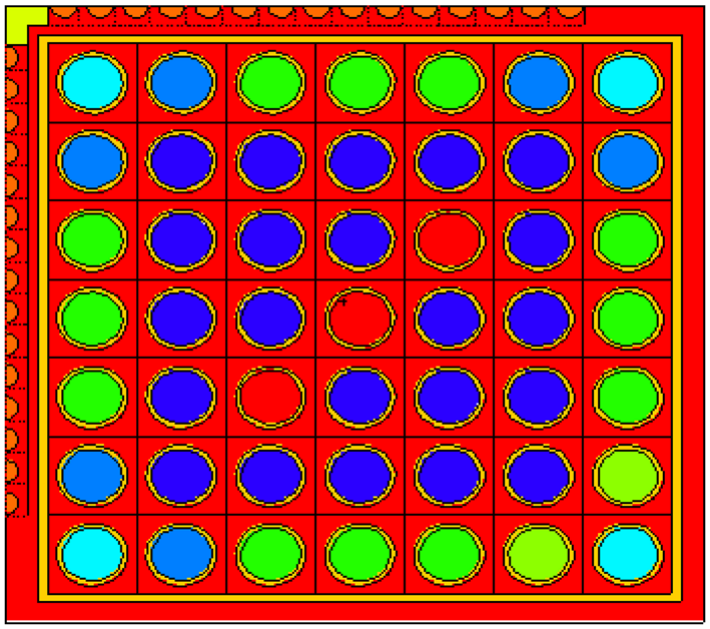
Enriched gadolinium bundle modeled by MCNPX code.

**Figure 3:**
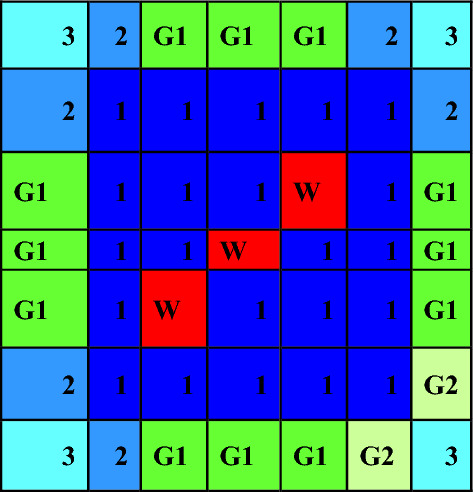
Fuel distribution in Gd bundle.

For gadolinium design, the average enrichment in the assembly is 17.9% U-235. The assembly contains 14 enriched gadolinium rods with an average enrichment of 12.4%. The reference fuel design performed by the MCNPX code is shown in Fig. 2. The fuel distribution and enrichment are depicted in Fig. 3 and Table 3 respectively. The fuel rods with enriched gadolinium are positioned at the assembly periphery (next to the water gap that surrounds the bundle) to suppress the large initial reactivity at the beginning of cycle and reduce the amount of residual poison at the end of cycle. The peripheral position of the gadolinium rods reduces their number within the bundle. This is attributed to the high thermal flux at the peripheral positions compared to the inner positions. Furthermore, the peripheral positioning of gadolinium rods contributes to enhancing the negativity of the void reactivity coefficient since the sensitivity of the void fraction tends to decrease. The enriched gadolinium composition is given in Table 4.

**Table 3:**
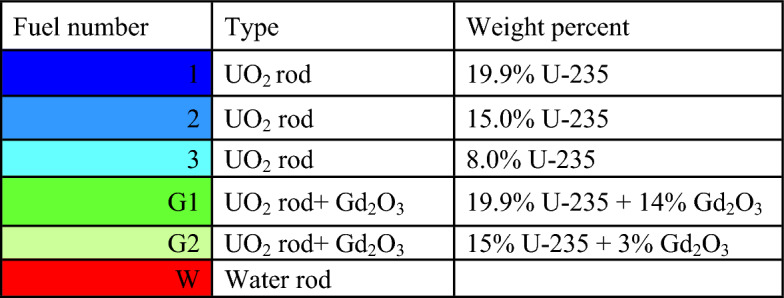
Fuel enrichment distribution in the bundle^21^.

**Table 4:** Isotopic composition of enriched gadolinium.

Isotope	Enriched Gadolinium (%)
Gd-152	
Gd-154	0.5
Gd-155	3.5
Gd-156	4.8
Gd-157	80
Gd-158	5.9
Gd-160	5.3

### Erbium reference design

**Figure 4:**
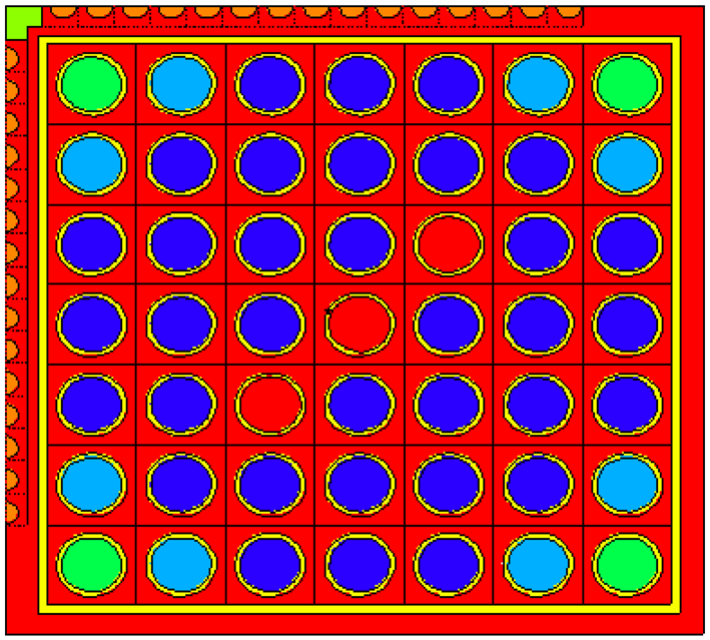
Enriched erbium bundle modeled by MCNPX code.

**Figure 5:**
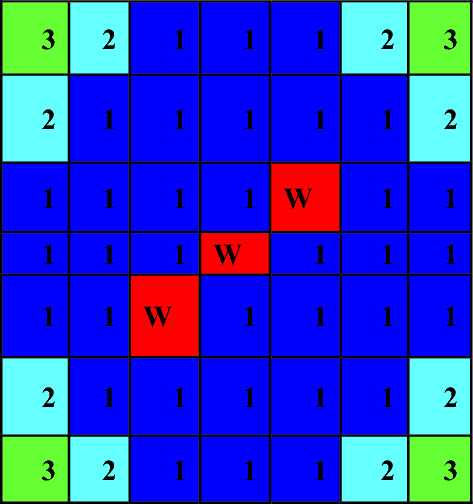
Fuel distribution in the Er bundle.

The model of the enriched erbium bundle is depicted in Fig. 4. The fuel distribution and enrichment are provided in Fig. 5 and Table 5, respectively. Because of its relatively low capture cross-section, erbium is mixed with UO_2_ in all fuel pins. Of all the erbium isotopes, Er-167 has the largest capture cross-section. Therefore, for this design, the erbium is enriched to 80% in Er-167 and the remaining percent, 20%, is set for Er-166. The average enrichment is also 17.9% U-235, which is the same as for gadolinium, allowing a fair comparison of the two cases. Based on the fuel distribution pattern, there are 40 fuel rods containing 3% erbium. The twelve fuel rods located at the bundle periphery are mixed with a small percentage of erbium (3% Er_2_O_3_) to compensate for the reduced U-235 enrichment.

**Table 5:**
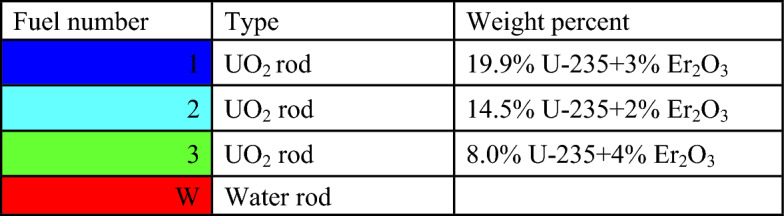
Fuel enrichment distribution in the bundle^11^.

### Natural boron design

**Figure 6:**
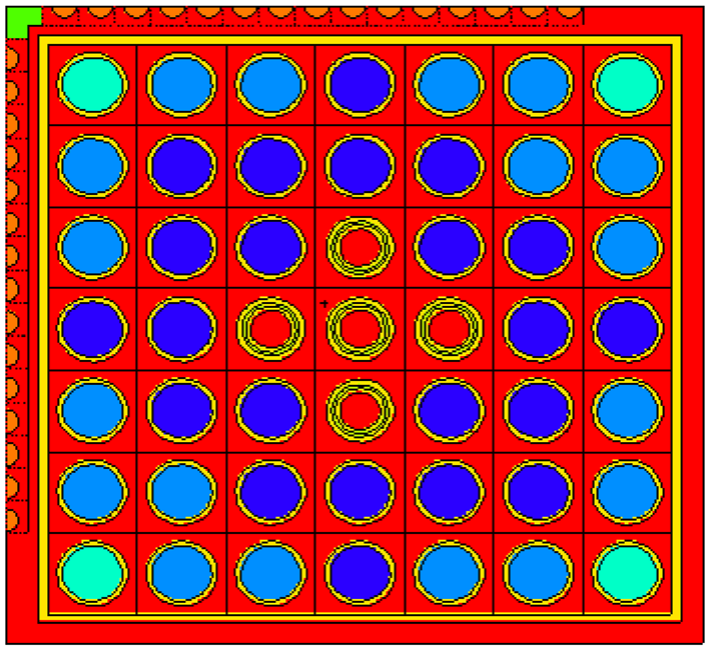
Enriched gadolinium bundle modeled by MCNPX code.

**Figure 7:**
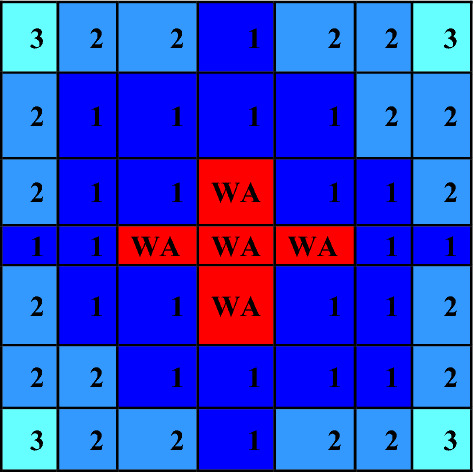
Fuel distribution in the bundle.

**Figure 8:**
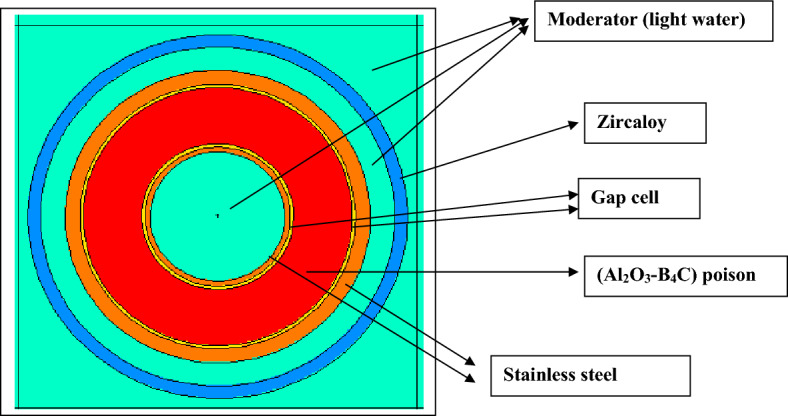
A schematic diagram of burnable WABA (Al_2_O_3_–B_4_C) modeled by MCNPX code.

The design of the natural boron bundle is shown in Fig. 6. The fuel distribution and enrichment are depicted in Fig. 7 and Table 6, respectively. The average enrichment of this bundle is 14.3%, which is lower than that of gadolinium and erbium bundles. As seen in Fig. 6, the design employs 44 fuel rods and 5 rods containing B_4_C in alumina. The annular design of WABA is illustrated in Fig. 8.

**Table 6:**
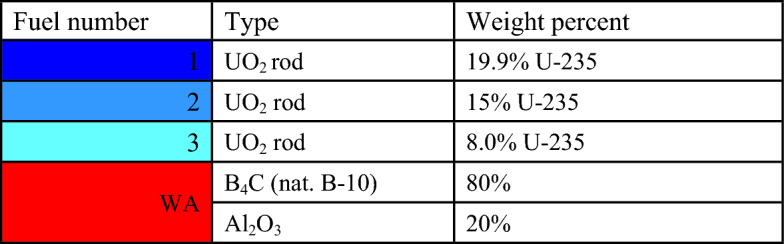
Fuel enrichment distribution in the bundle^11^.

Since this design has fewer fuel rods (44) than the other two designs (46), it has a higher average rod power. Therefore, the local peaking factors are multiplied by the rod power normalizing factor (46/44 = 1.045) so that they can be compared based on their actual rod power.

## Method of calculation

In this work, all the BWR bundle calculations were performed with the well-known Monte Carlo radiation transport computer code, MCNPX. This code a Fortran90 Monte Carlo radiation transport computer code that is based on the combination of CINDER90 and Monte Burns. The code version used in this work is MCNPX 2.7. The new capabilities of this version are used to analyze the effect of enriched gadolinium, enriched erbium, and natural boron on the safety parameters of BWR assembly. The infinite multiplication factor, power distribution, and the masses of fuel and poison isotopes are among these parameters that are estimated and analyzed at the various stages of fuel irradiation^25^.

The MCNPX simulation was run at (nps) 50,000 neutrons, 50 skipped cycles, and 250 active cycles. This results in a standard deviation in the output file ranging from 0.00032 to 0.00037 for all the K-inf results. The relative error parameter should examined in the output file, because it is very important for deciding the acceptability of the tally results. This parameter is calculated by the MCNPX code via dividing the standard deviation of the tally mean to the mean. In the current results, the relative error is found to be less than 0.1, which indicates that the tally results are reliable. This matches with the confidence percentage established by the manual^26^. The tallies used in this work are f4, fm4, and sd4 to calculate the power distribution (MW).

The infinite multiplication factor is calculated using the K code, which is formulated as K code 50,000 1 50 250, as stated before. The burn card must be set for calculating the masses of fuel and poison isotopes at the different stages of burnup. This card requires the following parameters:

(i) The bundle power, which is equal to $\left( {\frac{{900 \,\,MW \left( {Core \;thermal \;power} \right)}}{{ 956\,\, \left( {Total \;number \;of \;fuel \;asemblies} \right)}}} \right)$ = 0.94 MWatt
(ii) The time steps
(iii) The power fraction, which is taken to be 1 for all time steps
(iv) The materials that undergo burnup and their volumes.

The power distribution is calculated using the following cards

(i) F4 that estimates the flux in units (1/cm^2^),
(ii) Sd4 that expresses the volume of the material undergoing depletion (cm^3^)
(iii) The multiplier card, fm4 that takes the formula

fm4 (Normalized factor material number -6 -8). The normalized factor is taken to be 1, -6 represents the total fission cross section (barn) and -8 refers to the energy per fission (MeV/fission).

## Results and discussions

### Validation of MCNPX code with CASMO-4 code for gadolinium and erbium designs

Firstly, the MCNPX code is validated with CASMO-4^11^. This is carried out by comparing the reactivity values obtained by the MCNPX code with those predicted by CASMO-4 results at 40% void. CASMO-4 is a multi-group two dimensional transport code developed by Studsvik. It is one of the deterministic lattice codes, which is capable of simulating BWRs and PWRs at the assembly and unit cell levels. It also can generate two group cross sections and many neutronic parameters for the fuel assembly models. Modeling geometries consisting of cylindrical fuel rods positioned in a square or hexagonal lattice can be performed by this code^27^.

**Figure 9:**
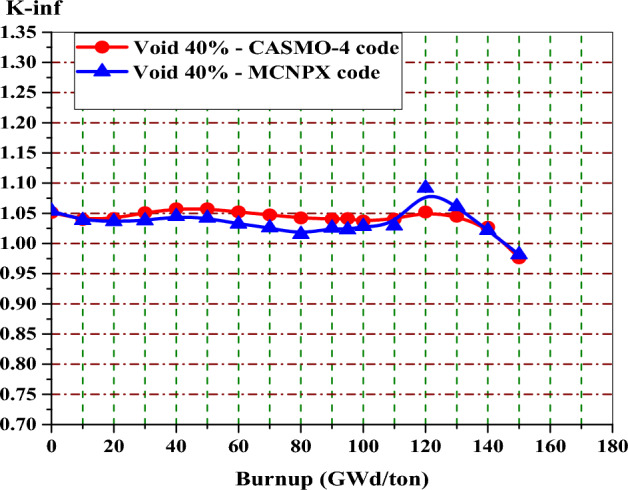
K-infinity versus exposure for enriched gadolinium bundle at 40% by MCNPX and CASMO-4 codes.

As seen in Fig. 9, a good agreement is found between the two code results for the gadolinium design. The maximum percent difference relative to the CASMO-4 code is 3.6% at 120 GWd/ton. Meanwhile, at 10 GWd/ton, the minimum relative percent difference is 0.15%.

In Fig. 9, one can see that the reactivity peak obtained by MCNPX code at 120 GWd/ton is higher than that obtained by CASMO-4 code. This means that the depletion of gadolinium is slower in the case of CASMO-4 model. The reason for this slow depletion of gadolinium is the number of Gd-Fuel regions modeled in the geometry. As the number of Gd-fuel regions increases, gadolinium fuel depletes more slowly. This in turn causes a small variation of K-inf with burnup owing to decreasing the number of thermal neutrons reaching the inner fuel regions (Shadowing effect of gadolinium). In CASMO-4, the gadolinium-fuel is divided into equal five regions. But, only three fuel regions are modeled by the MCNPX code for minimizing the time of calculation. As a result, the depletion of gadolinium is faster in the case of MCNPX model.

**Figure 10:**
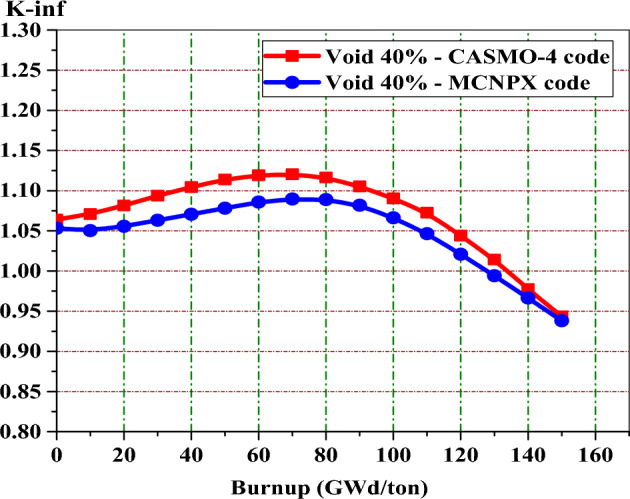
K-infinity versus exposure for enriched erbium bundle at 40% by MCNPX and CASMO-4 codes.

For the reactivity comparison in the erbium design, it is found that the maximum and minimum relative percentage differences are 3.2% and 0.56% at 50 and 150 GWd/ton, respectively. The CASMO-4 results are higher than those of MCNPX because of some fission products that are taken into consideration in the MCNPX simulation. These products are responsible for lowering K-inf during the fuel burnup. This is illustrated in Fig. 10.

### Analyzing the effect of 40% and 80% voids on the reactivity, peaking factor, build up of Pu-239, thermal neutron fraction and fissile inventory ratio for enriched gadolinium bundle

In this section, the author discusses the effect of 40% and 80% void fractions on the fuel performance of the BWR Gd-design. Generally, these void fractions may result from the boiling of the moderator or the loss of coolant in the reactor core. The 80% void is a theoretical void fraction used only for understanding and analyzing the impact of increased void fractions on the depletion calculations of the proposed designs under consideration. Based on these neutronic calculations, the burnup stage at which K-inf reaches its highest value can be determined at each void state. Determining the peak reactivity point is important since it expresses the exposure value at which all gadolinium content in the bundle is almost depleted.

**Figure 11:**
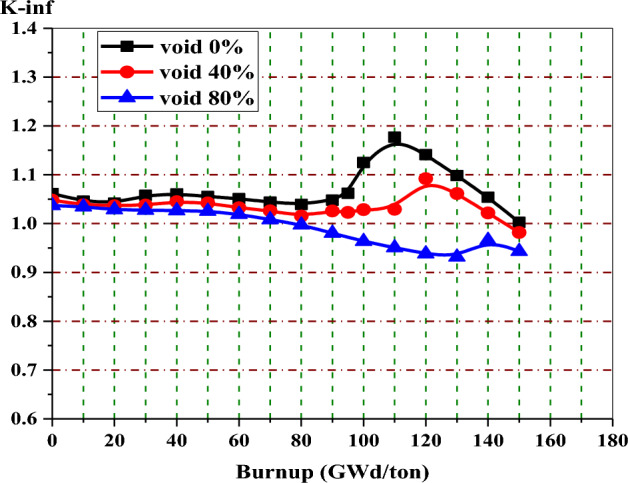
K-infinity versus exposure for enriched gadolinium bundle at different voids.

Figure 11 shows the behaviour of K-inf with burnup by MCNPX code at void fractions 0%, 40%, and 80%. The three curves have the same behavior, but they are different in the y-location of the reactivity peak. It is clear that at 0% void (the moderator density increases), the moderation increases, and hence more fission events occur. This results in a higher K-eff. The burnup value corresponding to the reactivity peak at 40% voids is greater than that corresponding to the reactivity peak at 0% void.

At 40% void, the K-eff reaches a maximum value at 120 GWd/ton, at which all gadolinium content is exhausted. After 120 GWd/ton, the K-eff starts to decrease linearly towards the end of life due to the depletion of the remaining U-235 content. At 40% void, the 15 years of operation is achieved at 113 GWd/ton by the gadolinium design.

**Figure 12:**
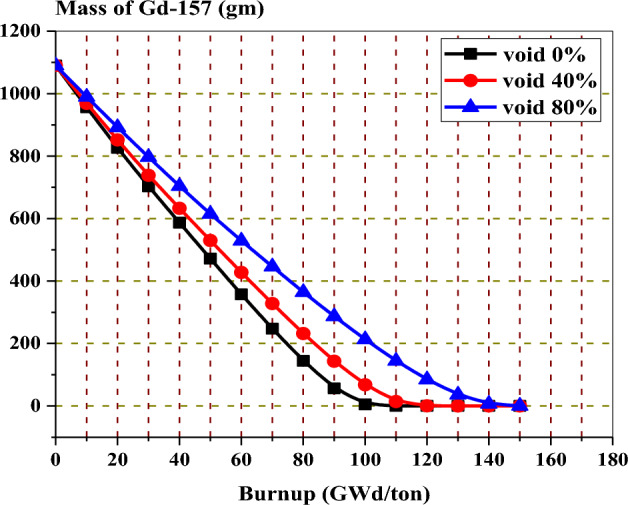
The mass of gadolinium-157 versus exposure bundle at different void fractions.

The variation of Gd-157 mass with exposure at 0%, 40%, and 80% void fractions is illustrated in Fig. 12. The behaviour shows that the Gd-157 decreases with fuel burnup till it reaches a constant concentration at 110, 120, and 140 GWd/ton for 0%, 40%, and 80% voids, respectively. These constant burnup values are the same as the values at which the three peak reactivities occur, as shown in Fig. 11.

The variation of the void reactivity coefficient with fuel depletion depends mainly on the U-238 concentration that causes neutron spectrum changes. As the burnup proceeds, the concentration of U-238 decreases marginally and the amount of fissile isotopes decreases. This will lead to an increase in the thermal neutrons that will be available to be absorbed by U-238. Therefore, the neutron spectrum will be much harder during voiding^28^.

**Figure 13:**
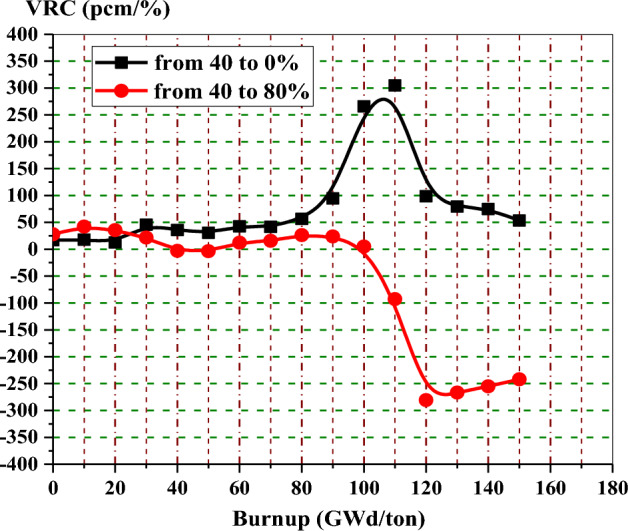
The void reactivity coefficient versus exposure for natural gadolinium bundle at different voids.

Figure 13 shows the void reactivity coefficient in pcm/% of enriched gadolinium reference fuel as a function of burnup. The upper curve shows the reactivity increase for the majority of the time, owing to the instantaneous change from 40 to 0% void. The bottom curve depicts that the negativity of VRC increases with time when the void percent changes from 40 to 80%. The positive reactivities at the low exposures in the two curves do not cause a problem during the actual reactor operation because the core would be provided with control rods at these exposures. Void reactivities for the two cases saturate after 120 and 130 GWd/ton when gadolinium-155 and 157 are completely depleted.

**Figure 14:**
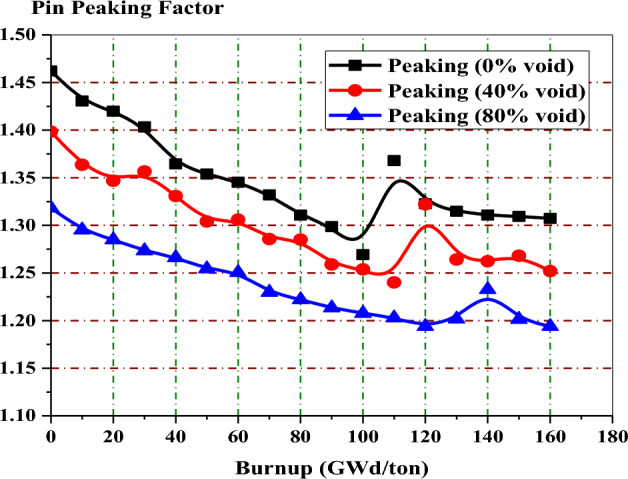
The local peaking factor versus exposure for enriched gadolinium bundle at different void fractions.

Figure 14 depicts the variation of peaking factor with burnup for gadolinium bundle at 0%, 80% and 40% void fraction. In all void cases, the peaking factor decreases with burnup without fluctuations and then increases at the exposure value at which the gadolinium is depleted.

The local peaking factor at 0% void is very high (1.46) at the beginning of life (BOL). This is attributed to the black neutron absorber, Gd-157, that tends to absorb thermal neutrons at the assembly periphery. Therefore, the power produced by the inner fuel pins is higher than that produced by the edge fuel pins. Furthermore, the presence of the three water holes in the centre of the assembly contributes to increasing the power due to its moderation effect. As a result, the local peaking factor is high and located around the guide tubes at the middle of the bundle, as we will see in the power distribution section.

**Figure 15:**
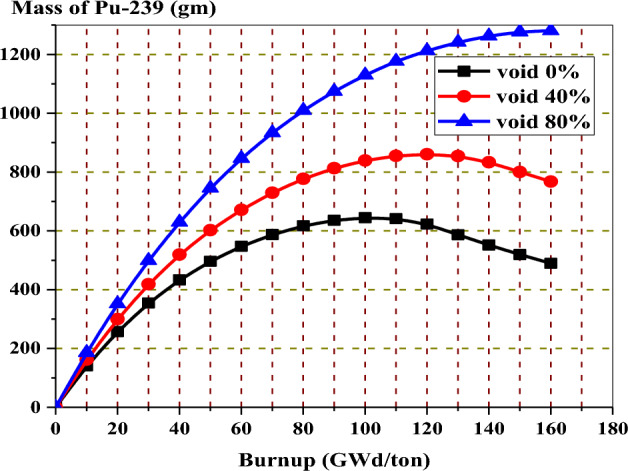
The mass of Pu-239 versus exposure for enriched gadolinium bundle at different void fractions.

The depletion of Pu-239, the fissile inventory ratio, and the thermal neutron fraction are also affected by the variation of the void fraction. As well known, as the void percent increases, the density of water decreases, and its ability to thermalize fast neutrons decreases. As a consequence, the number of thermal neutrons decreases, and a large fraction of fast neutrons will be available to be captured by U-238 to produce Pu-239. For this reason, the concentration of Pu-239 will be greater in the case of 80% void, as illustrated in Fig. 15.

**Figure 16:**
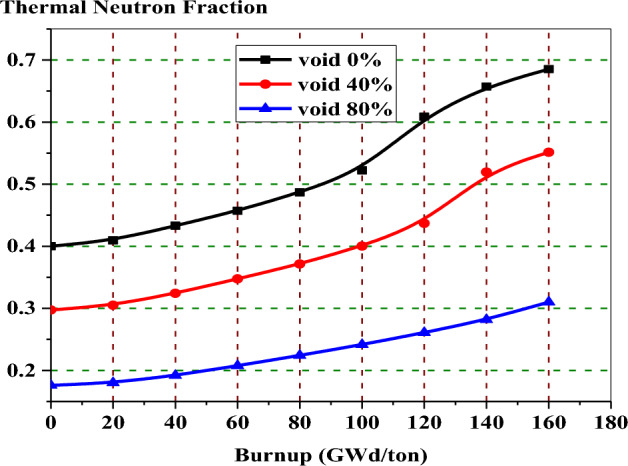
The thermal neutron fraction versus exposure for natural gadolinium bundle at different void fractions.

Figure 16 presents the thermal neutron fraction against the burnup steps for natural gadolinium bundle at 0%, 40%, and 80% void states. It can be seen that the reduced neutron moderation (higher void) due to the void formation causes the hardening of neutron flux. The reason for flux hardening during voiding is the increase of fast and resonance neutron fluxes while decreasing the thermal neutron flux.

**Figure 17:**
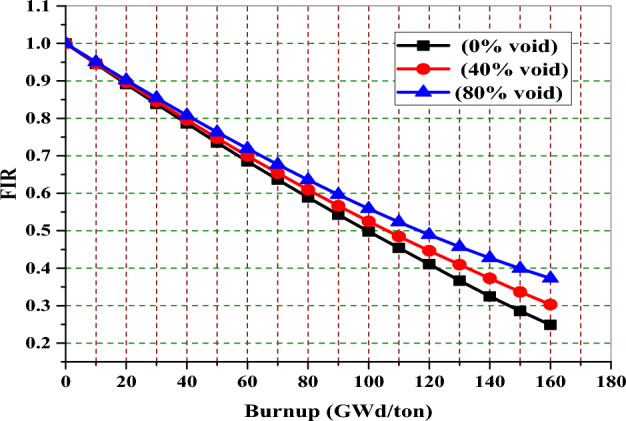
The Variation of the FIR versus exposure for natural gadolinium bundle at 0% and 40% void fractions.

Figure 17 depicts the variation of fissile inventory (FIR) ratio with burnup. This parameter (FIR) can be defined as the fissile inventory at a specific time divided by the initial assembly fissile inventory. It can be calculated through Eq. (1):

1 $$ {\text{FIR}}\;{ = }\;\frac{{{\text{N}}_{{{\text{U - 235}}\left( {\text{t}} \right)}} {\text{ + N}}_{{{\text{Pu - 239}}\left( {\text{t}} \right)}} {\text{ + N}}_{{{\text{Pu - 241}}\left( {\text{t}} \right)}} {\text{ + N}}_{{{\text{Np - 239}}\left( {\text{t}} \right)}} }}{{{\text{N}}_{{{\text{U - 235}}\left( {0} \right)}} {\text{ + N}}_{{{\text{Pu - 239}}\left( {0} \right)}} {\text{ + N}}_{{{\text{Pu - 241}}\left( {0} \right)}} {\text{ + N}}_{{{\text{Np - 239}}\left( {0} \right)}} }} $$

Where N fissile (t) refers to the atom density of the fissile isotope at a certain time (t) and N(t) fissile refers to the atom density of the fissile isotope at zero burnup stage (t = 0 days). As can be seen from Fig. 17, the values of FIR decrease with burnup for the three void cases due to the consumption of U-235 with time. It can be noticed that FIR values at 80% void are higher than those at 0% void. This is attributed to the lower thermal effect of the moderator when its density is decreased (void is increased). As a result, the masses of U-235, Pu-239, Pu-241, and NP-239 are higher at 80% void, particularly at the later stages of burnup.

### The depletion calculations for enriched erbium design

**Figure 18:**
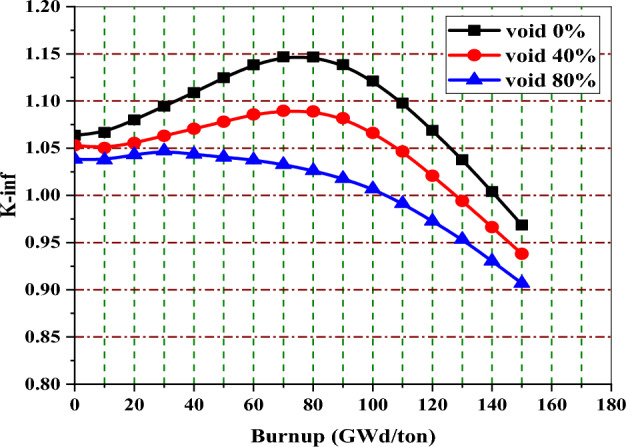
K-infinity versus exposure for enriched erbium bundle at different void fractions.

The K-infinity change versus burnup is shown in Fig. 18 for the erbium design. The K-inf swing of this design is larger than that of enriched gadolinium.

This is due to the smaller capture cross-section of erbium compared to gadolinium. For 40% void, 113 GWd/ton corresponds to 15 years of operation in that design.

**Figure 19:**
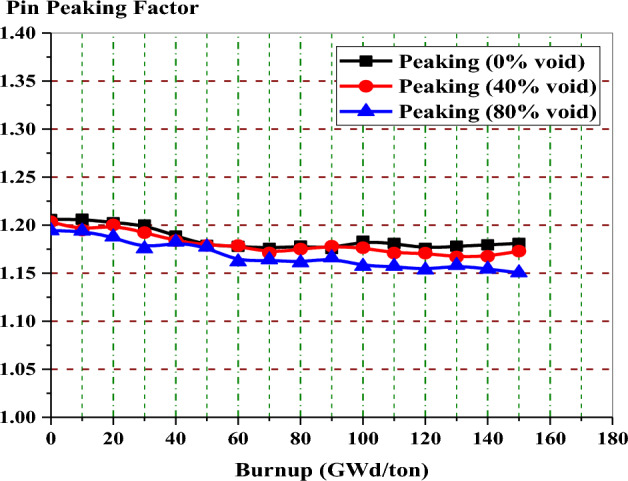
Local peaking factor versus exposure for enriched erbium bundle at different void fractions.

Figure 19 shows that the pin peaking factor is lower than that of the gadolinium case at all the burnup stages. Such behavior is expected due to the lower capture cross-section of erbium. This necessitates loading erbium uniformly in all fuel pins in the assembly (46 pins). This uniform loading makes the local power distribution relatively flat over time.

**Figure 20:**
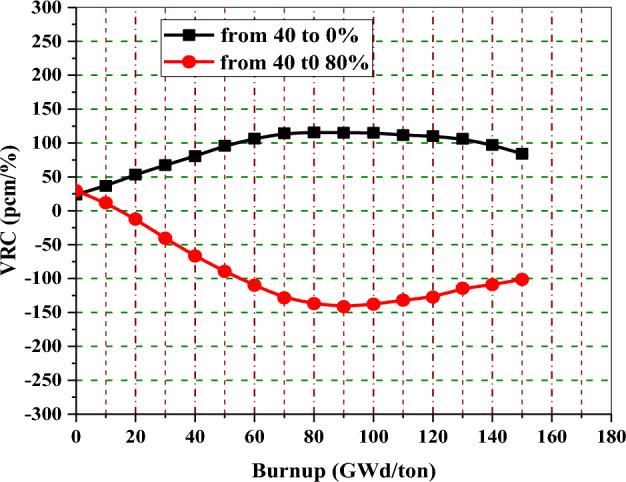
The void reactivity coefficient versus exposure for enriched erbium bundle at different void fractions.

Figure 20 provides the void reactivity coefficient of the enriched erbium bundle as a function of fuel burnup. The upper curve depicts that there is an increase in void reactivity for the majority of the time because of the transition from 40 to 0% void. The bottom curve depicts the increase of negativity of VRC with time when the void percent changes from 40 to 80%. Positive void reactivities at the low burnup stages are not a problem during reactor operation because control rods can be inserted into the core to reduce reactivity at these stages.

### The depletion calculations for natural boron reference design

In this design, the author carried out many trials by MCNPX code for positioning the WABA rods. These trials aimed to achieve a small K-inf swing over the life time of operation. This was accomplished when five WABA rods were positioned throughout the entire assembly.

**Figure 21:**
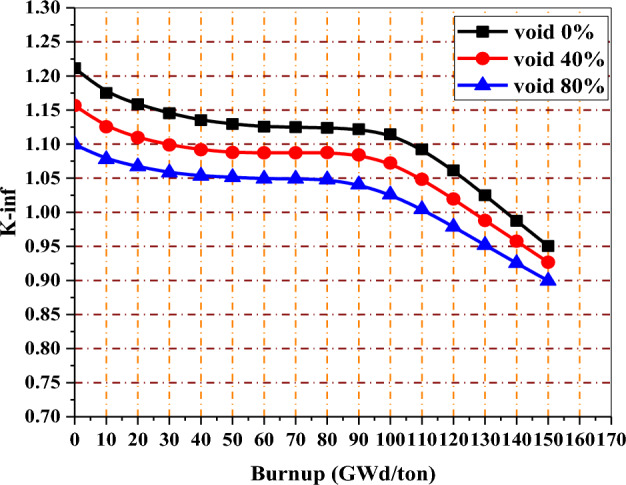
K-infinity versus exposure for natural boron bundle at different void fractions.

By this method, the fuel pins neighbouring WABA will deplete more slowly owing to the absorption cross section of boron that absorbs most of the thermal neutrons in the central region. As a sequence, the thermal neutrons flux are reduced towards the bundle periphery. After that, these fuel pins will start to burn when the amount of boron in the B_4_C rods is depleted. As we can see in Fig. 21, the K-inf is high at the beginning of life and stabilizes from 30 to 100 GWd/ton, with a very small swing at this exposure range. The modeling of WABA rods used 20% B_4_C (20% B-10 and 80% B-11) and 80% Al_2_O_3_. Extending the fuel cycle length to 15 years is reached at 114 GWd/ton for 40% void.

**Figure 22:**
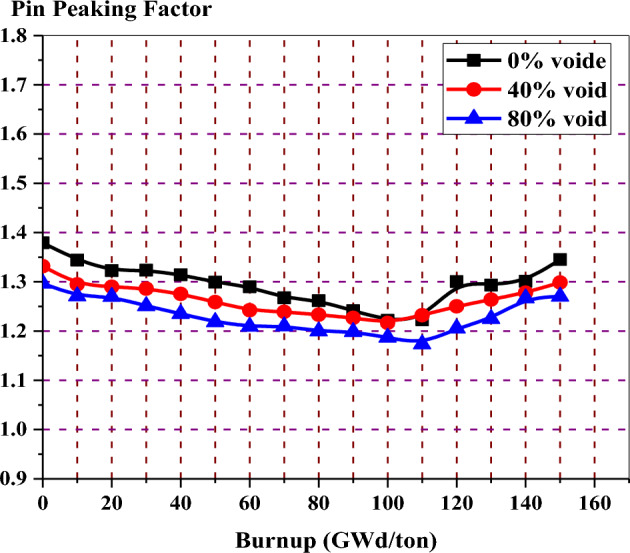
Local peaking factor versus exposure for natural boron bundle at different void fractions.

Figure 22 shows the local peaking factor with burnup for the natural boron bundle. In this design, there are 45 fuel pins instead of 46 as in the gadolinium and erbium designs. To obtain the same average heat flux of gadolinium and erbium designs, the peaking factor of natural boron design must be standardized. This is carried out by multiplying the B_4_C peaking factor by 1.022 (46/45).

**Figure 23:**
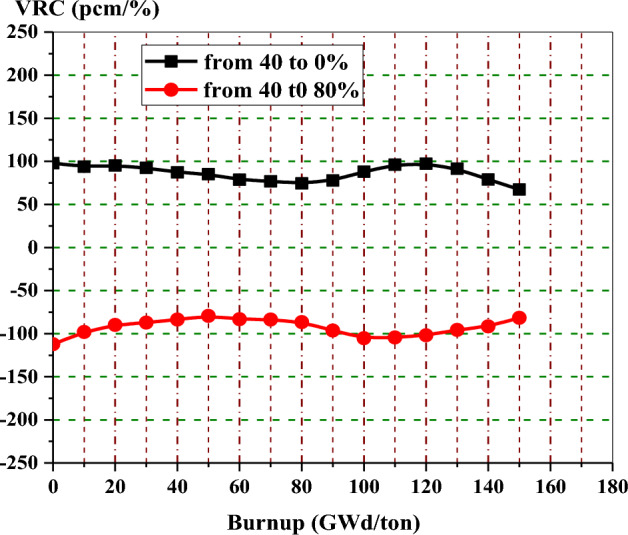
The void reactivity coefficient versus exposure natural boron bundle at different voids.

Figure 23 shows void reactivity for the natural boron model as a function of bundle exposure. It can be noticed that the void reactivity is quite constant throughout the whole exposure range. The maximum value of void reactivity for an enriched gadolinium bundle is about 3 times the constant relativity value obtained in the B_4_C design. But during the majority of the cycle, the B_4_C design presents larger void reactivities than the enriched gadolinium.

### A comparative study of the three models

First of all, the power distribution for the three BWR designs at 40% void is presented and analyzed. This is necessary for determining the position of the peaking factor in each bundle and studying how the power is distributed in each design based on the type of the burnable poisons.

Tables 7, 8 and 9 illustrate the power distribution (MW) across gadolinium, erbium and boron bundles respectively at the beginning of life (BOL). There are common characteristics in the three bundles as,

**Table 7:**
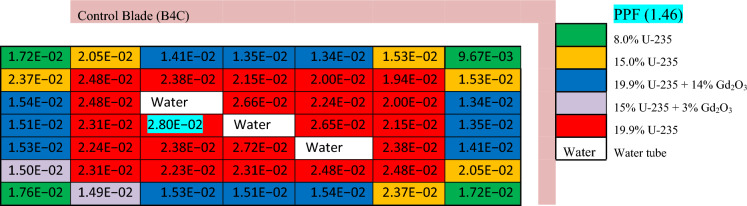
The power distribution (MW) in enriched gadolinium bundle at BOL.

**Table 8:**
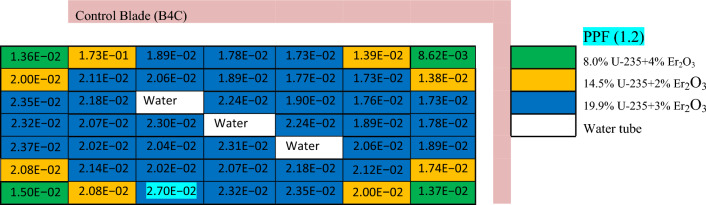
The power distribution (MW) in enriched erbium bundle at BOL.

**Table 9:**
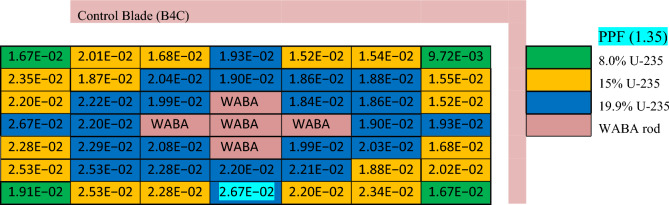
The power distribution (MW) in natural boron bundle at BOL.

(1) As water converts the fast neutrons into thermal neutrons, the power produced from the fuel rods located around the water tubes is greater than that of those located at the assembly periphery. (2) The insertion of the B_4_C control blade has a remarkable effect on decreasing the power at the right and upper parts of the assemblies. This is expected, owing to the high absorption cross section of B-10, which reduces the number of thermal neutrons that can reach these fuel pins. (3) The fuel pins located at the left and lower parts of the assembly have slightly higher power values due to the water gap around the assembly.

In Tables 7 and 8, it is noticed that there is a remarkable difference between the power values at the centre and at the periphery. The main reason for this difference is the presence of high concentrations of gadolinium and erbium at the bundle periphery (next to the water gap around the bundle). Accordingly, the power results are reduced at these positions compared to those at the centre. This peripheral position of Gd and Er is favourable for preventing the positive void reactivity coefficient since the sensitivity of the void fraction gets smaller. This ensures that this remarkable difference is required to enhance the negativity of the void reactivity coefficient.

In the enriched gadolinium design, one can see that the pin peaking factor (1.46) is located in the fuel pin next to the water tube in the central part of the assembly. But for enriched erbium design, the peaking factor (1.2) is found in the fuel pin, with 19.9% enrichment at the lower part of the assembly. This is predictable due to the high enrichment in that pin and the presence of a water gap around the bundle. Finally, for natural boron design, the peaking factor (1.35) is located at the left side of the assembly in the fuel pin with an enrichment of 19.9% for the same reasons that are stated in the erbium case.

**Figure 24:**
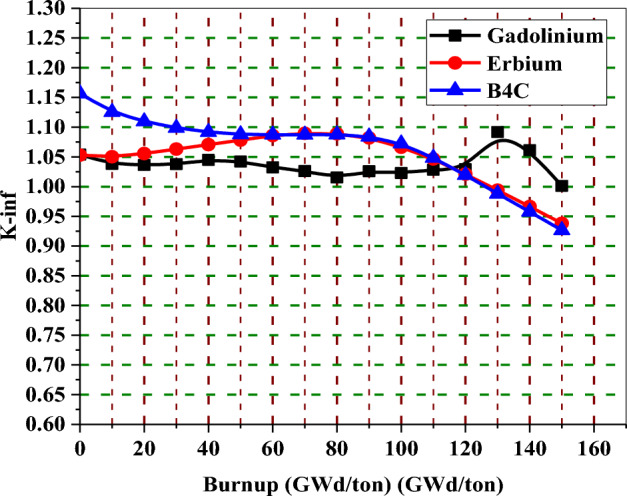
K-inf with exposure for the three models at 40% void.

In Fig. 24, the K-inf swing for the enriched gadolinium bundle is clearly the smallest. On the other hand, the erbium bundle has a higher K-infinity swing, where the K-inf is peaked at about 70 GWd/ton. The B_4_C model presents the highest reactivity values during the early stages of fuel irradiation despite the lower average enrichment contained in that model. This is due to the thermalization effect originating from the strategic placement of (Al_2_O_3_–B_4_C) rods rods inside the assembly.

**Figure 25:**
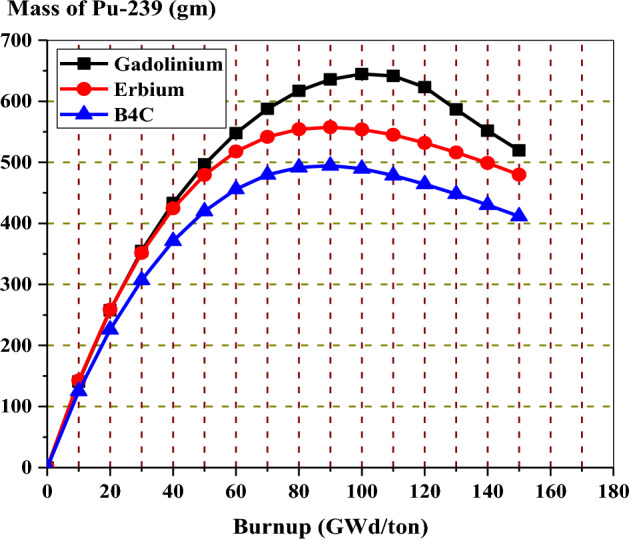
Mass of Pu-239 with exposure for the three models at 40% void.

In the analysis of K-inf variation versus burnup for the three models, it is clear that the rate of reactivity loss at high burnups is similar but not identical for all burnable absorbers. As seen in Fig. 24, the reactivity loss is greater for the B_4_C design compared to gadolinium or erbium designs. This could be attributed to the high moderator to fuel ratio, which limits the resonance capture in U-238. As a result, as illustrated in Fig. 25, plutonium production is reduced.

**Figure 26:**
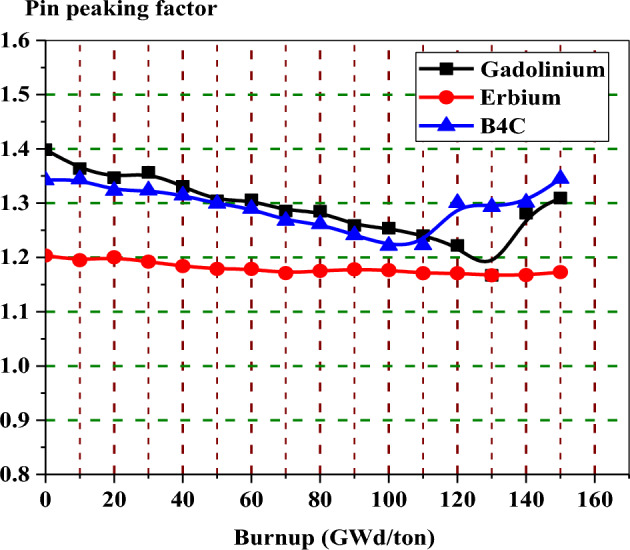
K-inf with exposure for the three models at 40% void.

For local peaking factor comparison in Fig. 26, the gadolinium design has the highest peaking factor at the beginning of life (BOL). The erbium design presents a flattening behaviour of the peaking factor at all the burnup steps. This is due to the uniform distribution of erbium in all fuel rods in the assembly. Therefore, the erbium model is found to be superior in terms of the power peaking factor.

The peaking factor behaviour of the boron design comes after that of the gadolinium design due to the dual function of B_4_C–Al, which acts as a neutron absorber and moderator. This can be explained by the fact that B_4_C–Al has the ability to thermalize fast neutrons into thermal neutrons that will be available for the interaction with U-235 in the fuel rods located behind B_4_C–Al. This will contribute to producing more power at the edges.

**Figure 27:**
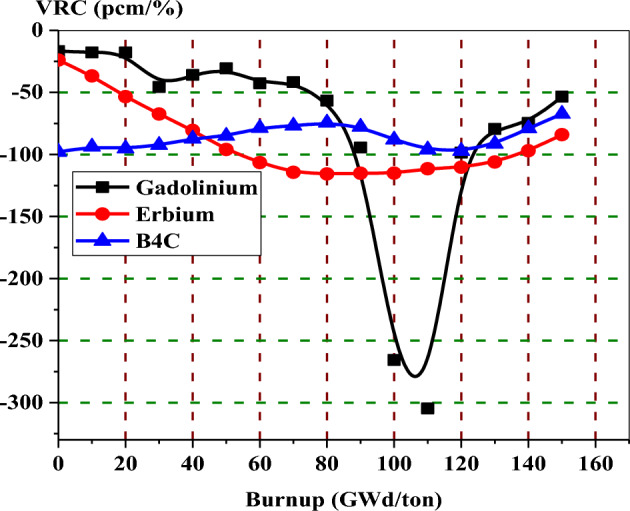
The void reactivity coefficient versus exposure for the three models at changing void from 40 to 80%.

Figure 27 provides the void reactivity coefficients with burnup for the three bundles when the void fraction increases from 40 to 80%. All void reactivities for these assemblies saturate after about 120 or 130 GWd/ton. For the assembly loaded with B_4_C–Al as an aburnable absorber, void reactivity remains quite constant over the full exposure range compared to erbium and gadolinium assemblies. This is due to the positions of B_4_C–Al pins within the assembly and the low absorption cross section of B-10 compared to Gd-155 and Gd-157. Furthermore, this model (B_4_C) has the most negative void reactivity coefficients, up to 40 GWd/ton, among all the models.

The gadolinium assembly delivers the lowest negative void reactivity coefficient up to 80 GWd/ton due to the highest neutron capture cross section and the location of the gadolinium rods at the edge of the assembly, and this results in high power values in fuel pins located in the entire assembly. On the other hand, the B_4_C model presents the highest negativity of VRC up to 40 GWd/ton. As the burnable absorbers are depleted towards the end of life, the assemblies loaded with erbium and B4C-Al exhibit a slightly more negative void reactivity than the gadolinium assembly.

**Figure 28:**
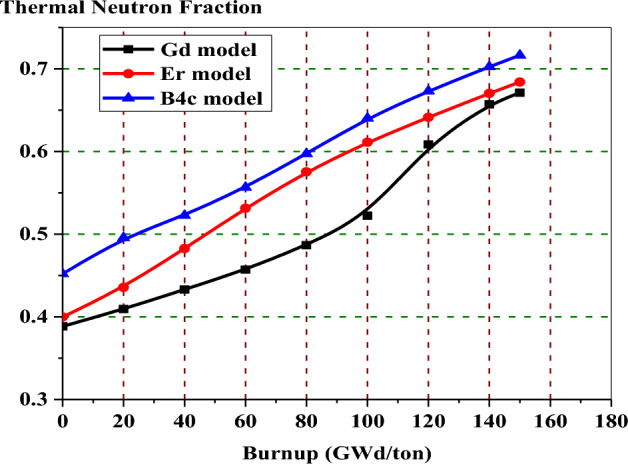
The thermal neutron fraction versus exposure for the three designs.

Figure 28 presents the thermal neutron fraction against the burnup steps for the three BWR bundles under investigation. It is observed that the neutron flux hardening is clearer in the enriched gadolinium model. This is due to increasing the fast and resonance neutron flux absorption by U-238. Also, it is clear that the thermal neutron flux of a boron design is larger than that of an erbium design due to the moderation effect of B_4_C-Al rods.

**Figure 29:**
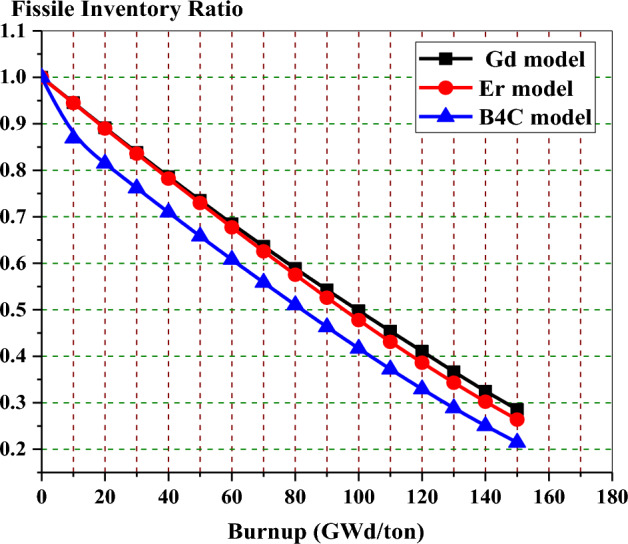
The fissile inventory versus exposure for the three models at 40% void.

**Figure 30:**
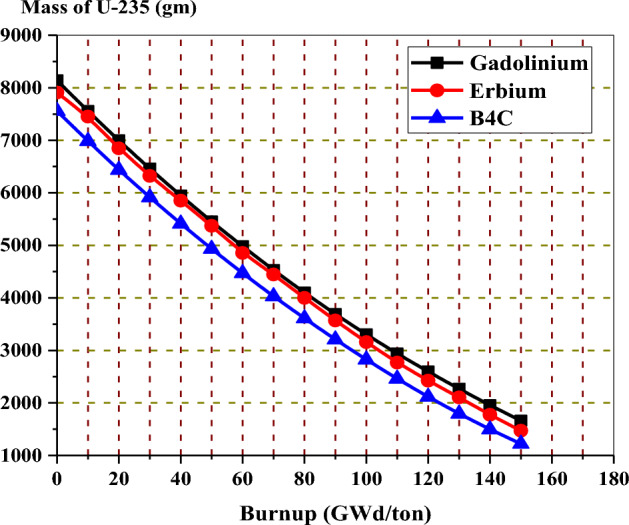
The variation of the mass of U-235 with exposure for the three designs.

Figure 29 shows that the natural boron design has the lowest fissile inventory ratio. This means that the U-235 content is lower for the natural boron design compared to the other two designs at the EOL. On the other hand, the Gd-model has the highest FIR of all models. The higher FIR contributes to achieving a longer core life and reducing the reactivity swing over the burnup steps. The depletion of U-235 in the three bundles is shown in Fig. 30. It is clear that the mass of U-235 decreases by 20%, 18.6%, and 16% of its initial value for gadolinium, erbium, and B_4_C models, respectively. This means that the B_4_C model contributes to saving U-235 content at the target exposure.

**Figure 31:**
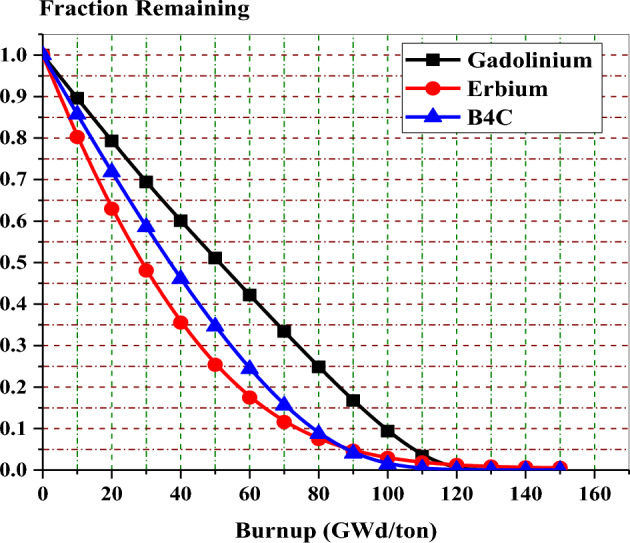
Fraction of burnable poison isotope with Burrup.

Figure 31 compares the fraction remaining for each burnable absorber at 40% void for the three designs. It can be observed from this figure that 95% of both Er-167 and B-10 are depleted at about 95 GWd/ton. And 95% of Gd-157 is burnt out at 110 GWd/ton. This means that the residual amount of poison is about 5% of the original amount. The figure also confirms the slow depletion of erbium compared to the other two absorbers. This explains the clear power flattening in the erbium design.

### The K-infinity behavior with and without the burnable poisons

Before studying this part, it is very important to interfere that keeping the nuclear reactor running without refueling for 150 GWd/ton (15 years) has been discussed by several researchers. Syarifah et al., have simulated (Uranium-Nitride and Plutonium Nitride) fuel in a gas cooled fast reactor for over 10 years of operation without refueling. This was conducted by utilizing high percentages of plutonium and specific radii and heights of loaded fuel in the reactor. They found that the best design of gas cooled fast reactor that can be operated for 10 years without refueling when the radius of F1:F2:F3 = 50 cm: 30 cm: 30 cm, the height of F1:F2: F3 = 50 cm: 40 cm: 30 cm and the plutonium percentage in F1:F2:F3 = 7%: 10%: 13% are employed in modeling the reactor core^29^. Alam et al., have performed whole core calculations on a small modular reactor to investigate the criticality of this reactor at least 15 years of operation with a reactivity swing of 400 pcm. They found that the PWR (without soluble boron) fueled with 15% and 18% U-235 can reach the criticality state at 16 and 17 years of operation for UO_2_ and micro heterogeneous ThO_2_–UO_2_, respectively^30^. Peakman et al.^31^, have also shown the possibility of extending the fuel cycle to 15 years of operation for PWR fueled with (Th–U)O_2_ without refueling. Barchevtsev et al.^2^, have obtained criticality at 100 GWd/ton when 19.8% U-235 is mixed with erbium in the pin cell geometry of a PWR. In the current research, long simplified boiling water reactor bundles are simulated by the MCNPX 2.7 code. The aim of designing such long simplified boiling water reactors is to eliminate the refueling machines and the fuel pool. This in turn improves the capacity usage ratio and reduces the need for spent fuel storage. The main disadvantage of long-cycle cores is that a large fraction of the fissile materials in the nuclear fuel remains un depleted, making the fuel cycle more expensive^11,20^.

In this part, the behavior of K-infinity with burnup before and after the implementation of burnable poisons is compared for the three proposed BWR bundles. The aim of this comparison is to demonstrate how the high initial reactivity is controlled in the presence of burnable poisons. Generally, the high fuel enrichment (average 17% U-235) causes high initial excess reactivity that should be decreased at the BOL to enhance the safety of the reactor. The addition of these absorbers not only suppresses the initial excess reactivity but also provides other several benefits, such as lowering peaking factors, flattening power distribution, increasing the negativity of FTC and MTC and reducing the K-inf swing.

**Figure 32:**
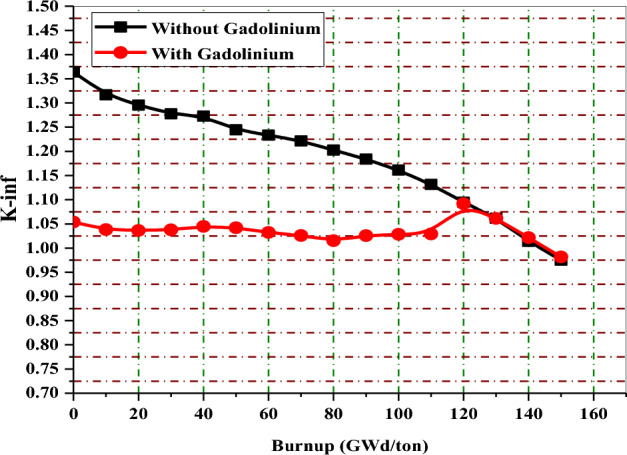
K-inf as a function of burnup with and without gadolinium.

**Figure 33:**
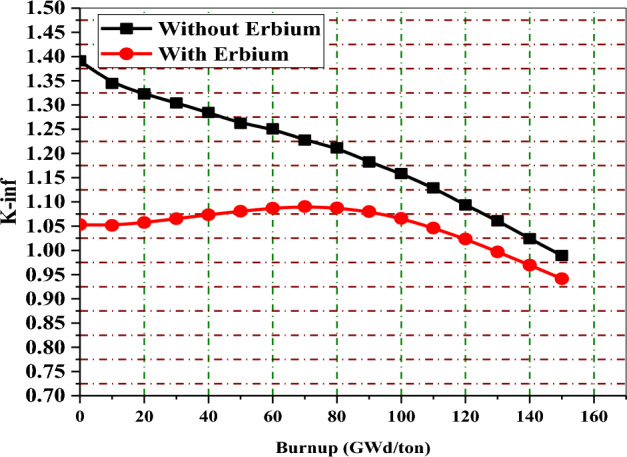
K-inf as a function of burnup with and without erbium.

**Figure 34:**
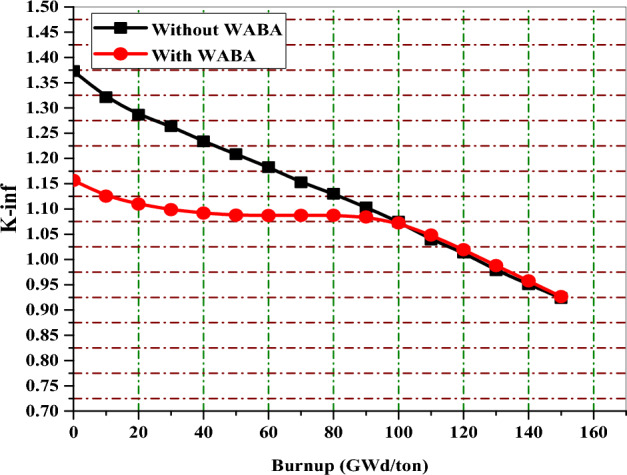
K-inf as a function of burnup with and without WABA rods.

Figures 32, 33 and 34 depict that the K-inf decreases linearly with exposure in the absence of the burnable absorbers. This is owing to the depletion of U-235 and the production of Xenon-135 and Sm-149, which act as important reactor poisons, because of their high capture cross sections. In the three designs with burnable absorbers, 15 years of operation are achieved at 111 Gwd/ton for gadolinium and erbium designs and at 113 GWd/ton for natural boron design. The designs without absorbers predict nearly the same burnup values corresponding to 15 years but with high initial excess reactivity.

This means that extending the time of reactor operation to 15 years with low initial excess reactivity (with absorbers) is a desirable feature to guarantee the safety of reactor operation.

### The FTC, MTC and CRW for the three designs

The fuel temperature coefficient, moderator temperature coefficient, and control rod worth are some of the most important safety parameters for light water reactors. From a reactor safety point of view, the reactor should run at negative FTC and MTC. On the other hand, the larger control rod worth provides a larger core shutdown margin.

To estimate the FTC, the fuel temperature is increased from 900 to 1200 K without changing the fuel density (the change in the fuel density is ignored). The FTC can be calculated using Eq. (2).

2 $$ {\text{FTC }}\;\left( {{\text{pcm}}/{\text{ k}}} \right) \, = \left( {\frac{{\user2{Kinf }_{{\user2{at }\,\,1200{\varvec{K}}}} \user2{ } - \user2{ Kinf }_{{\user2{at }\,\,900\user2{ K}}} }}{{({\varvec{Kinf}}_{{\user2{at }\,\,900{\varvec{K}}}} \user2{ * Kinf }_{{\user2{at }\,\,1200{\varvec{K}})}} }}} \right) $$

For MTC, the water temperature is lowered from 600 to 293.6 K. The density of water corresponding to each temperature is calculated. The temperatures of fuel and clad remain as they are. Equation (3) is used for calculating the MTC in units of (pcm/K).

3 $$ {\text{FTC }}\left( {{\text{pcm}}/{\text{ k}}} \right) \, = \left( {\frac{{\user2{Kinf }_{{\user2{at }\,\,600{\varvec{K}}}} \user2{ } - \user2{ Kinf }_{{\user2{at }\,\,293.6\user2{ K}}} }}{{({\varvec{Kinf}}_{{\user2{at }\,\,600{\varvec{K}}}} \user2{ * Kinf }_{{\user2{at }\,\,293.6{\varvec{K}})}} }}} \right) $$

The control rod worth is a crucial safety parameter that is influenced by many factors. These factors comprise fuel depletion, time of reactor operation and the position of control rods in the core. It can be calculated from Eq. (4).

4 $$ {\text{CRW }}\left( {{\text{pcm}}} \right) \, = \left( {\frac{{\user2{Kout } - \user2{ Kin}}}{{\user2{Kout* Kin}}}} \right) $$

where K_in_ is the multiplication factor value when the B_4_C control blade is inserted in the bundle. K_out_ is the multiplication factor when the B_4_C is replaced by C (Carbon) that contributes to increasing the core reactivity owing to its scattering cross section.

**Figure 35:**
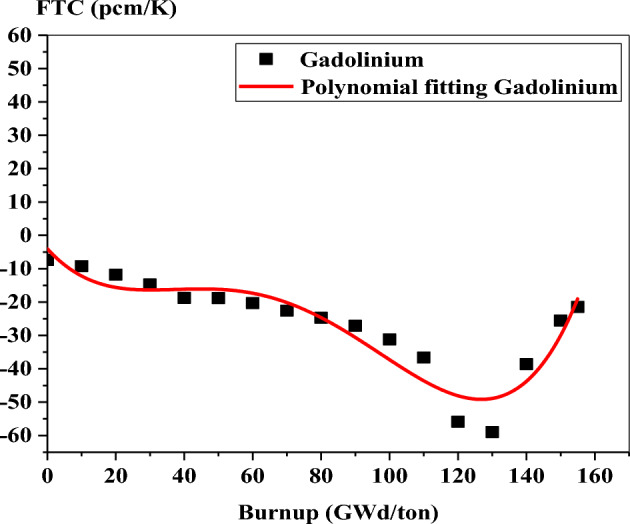
FTC as a function of burnup for gadolinium bundle.

**Figure 36:**
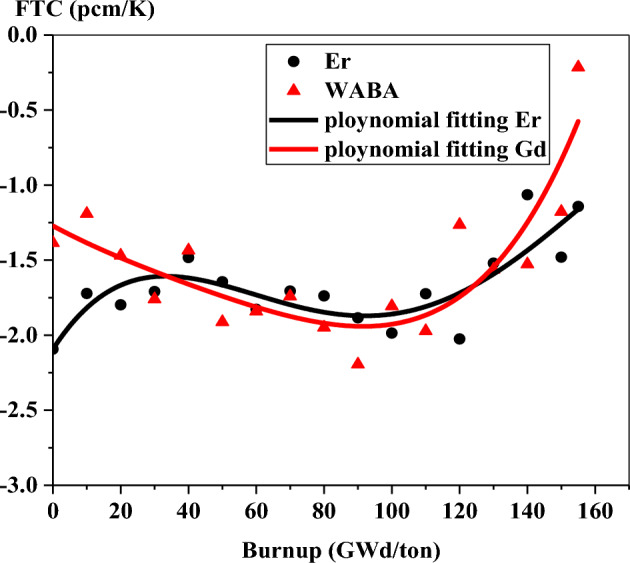
FTC as a function of burnup for erbium and WABA bundles.

Figures 35 and 36 show the variation of FTC with burnup for the three fuels. In the case of gadolinium fuel, the negativity increases with burnup till 130 GWd/ton, at which gadolinium is nearly completely depleted. After 130 GWd/ton, the negativity decreases with burnup because of the softening of the neutron spectrum after the consumption of gadolinium in the bundle. A comparable behavior of FTC is obtained for WABA design, but decreasing the negativity of FTC starts from 100 GWd/ton, at which B-10 is burnout.

At the BOL, the FTC is − 7.38 pcm/K, − 2.09 pcm/K and − 1.38 pcm/K for gadolinium, erbium and natural boron models, respectively. This means that the greater negativity of FTC is presented by gadolinium. The reason for that is the high capture cross section of Gd-157 compared to that of Er-167 and B-10. It is also noted that the FTC of Er-167 is more negative than that of B-10 due to the resonance peak absorption provided by erbium at 0.5 eV.

At the end of this part, it is worth noting that the nature of Monte Carlo method results in statistical fluctuations in the reactivity coefficients (FTC and MTC) with burup. Therefore, the application of polynomial fitting is recommended for the interpretation of the behavior of these coefficients.

**Figure 37:**
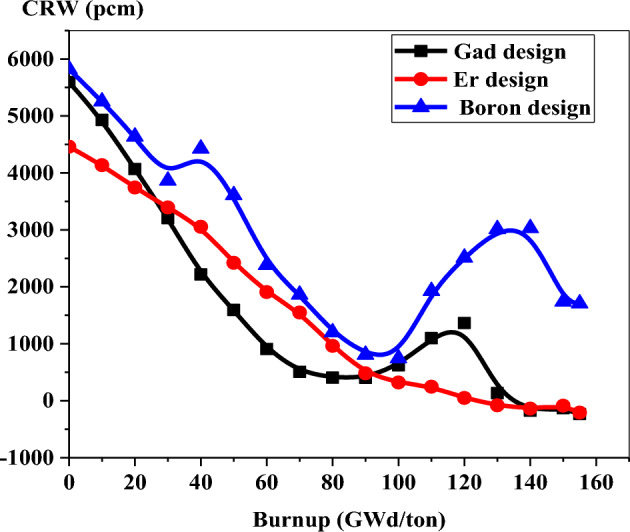
CRW as a function of burnup for the three designs.

As illustrated in Fig. 37, the control rod worth decreases with burnup for the three models, up to about 100 GWd/ton. The boron design provides the highest CRW values at all burnup stages. This is predictable because of the low capture cross section of B-10. The most interesting thing in the figure is that the CRW of erbium is lower than that of gadolinium at the early stages of burnup, in spite of the high capture cross section that gadolinium has. This is attributed to the uniform distribution of erbium that enhances the efficiency of capturing thermal neutrons. As a result, the CRW of erbium is lower. From 30 to 90 GWd/ton, the CRW values are higher for erbium compared to those of gadolinium. The control rod worth increase starting from 100 GWd/ton in the case of Gd at 100 GWd/ton because of the complete depletion of gadolinium at this burnup stage as shown in Fig. 12. As a result, the efficiency of capturing thermal neutrons by control rods increases.

**Figure 38:**
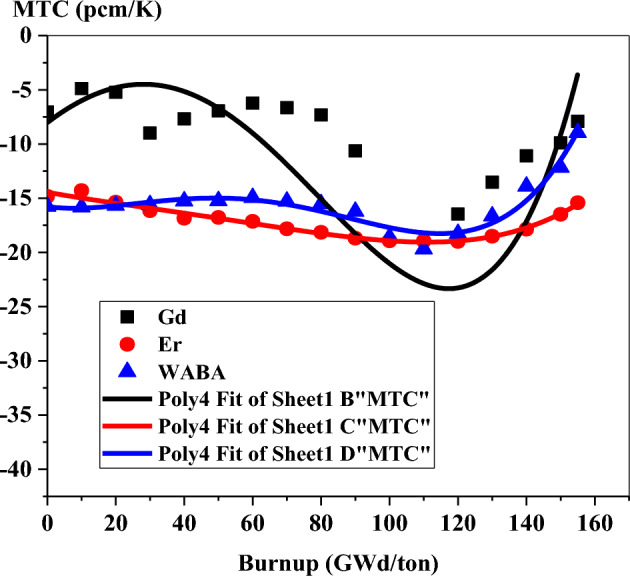
MTC as a function of burnup for the three designs.

Figure 38 shows that the negative value for MTC is increased remarkably at the BOL in WABA and erbium cases. The WABA design offers the most negativity of MTC till 20 GWd/ton. On the other hand, MTC is negative in the majority of time steps for the erbium design.

The large negative value of MTC in the case of the erbium model is owing to the resonance peak of Er-167 at 0.5 eV. Furthermore, the MTC of the gadolinium case is lower than that of the erbium and WABA cases. This is attributed to decreasing the thermal neutron flux that results from lowering the moderation process at increasing the moderator temperature. Nevertheless, even for the gadolinium model, the MTC remains negative till the EOL.

## Conclusion

Three BWR bundles with three different burnable absorbers are simulated by the MCNPX code. These absorbers included enriched gadolinium, enriched erbium, and natural boron. Some reactor safety features are investigated for the three designs at 40% void. These features involved power distribution, reactivity, local peaking factor, void reactivity coefficient, thermal neutron fraction, fissile inventory ratio, and the depletion of U-235. The MCNPX simulation showed that every design has a number of advantages over the others. This means that no one design is clearly superior. The conclusions of this work can be summerized in the following points.

- Based on the K-inf results with respect to the fuel exposure, it is clear that the cycle length of gadolinium and erbium bundles can be extended to 15 years at 113 GWd/ton. For the boron bundle, the cycle length can be extended to the same period (15 years) at 114 GWd/ton).
- In terms of reactivity, the use of enriched Gd-157 contributed to the best results with the smallest reactivity swing. The local peaking factor on the assembly level, however, was high (1.46) at 0 GWd/ton, compared to the erbium design, which has a peaking factor of about 1.2.
- Although erbium design displays more reactivity swing than the gadolinium design, the local peaking is the lowest of all three designs since erbium was loaded uniformly inside the assembly. This uniform loading makes the power distribution within the fuel assembly relatively flat throughout the life exposure.
- The natural born design has the advantage of preserving the U-235 inventory at the end of life. Furthermore, the reactivity flattens from 30 to 100 GWd/ton in this model. This is due to the slow depletion of fuel rods located nearby the WABA rods.
- FTC is more negative for gadolinium-uranium fuel because the Doppler broadening of fertile absorption is higher for that fuel when its temperature increases.
- The boron model offers the lowest CRW. This is expected due to the low capture cross section of B-10 in comparison with that of Gd-157 and Er-167
- The MTC of the gadolinium case is lower than that of the erbium and WABA cases. This is because of decreasing the thermal neutron flux that results from lowering the moderation process when the moderator temperature increases. This can be explained by the strategic placement of WABA rods and the uniform distribution of erbium which enhance the efficiency of thermal neutrons capturing till 80 GWd/ton.

## Data Availability

The data sets analyzed in the current study are available from the corresponding author on reasonable request.

## References

[1] Marcus GH (2000). Considering the next generation of nuclear power plants. Prog. Nucl. Energy.

[2] Barchevtsev V, Artisyuk V, Ninokata H (2002). Concept of erbium doped uranium oxide fuel cycle in light water reactors. J. Nucl Sci. Technol..

[3] Kryuchkov, E.*et al.* Fuel cycles with high fuel burn-up: Analysis of reactivity coefficients. In *Moscow Engineering Physics Institute (State University), Russia* (2003).

[4] Ramsdell Jr, J. *et al.* Environmental Effects of Extending Fuel Burnup above 60 Gwd/MTU. In *USNRC, Rockville, MD, NUREG/CR* 6703 (2001).

[5] Secker, J. Optimum cycle length and discharge burnup for nuclear Fuel: Phase 2: Results achievable with enrichments greater than 5 w/o. No. 1003217, EPRI (US) (2002).

[6] Blakely C, Zhang H (2019). Two-year PWR core design with burnup and enrichment extension using VERA-CS. Transactions.

[7] Stewart R, Blakely C, Zhang H (2021). Investigation of a two-year cycle pressurized water reactor core design with increased enrichment and extended burnup limits. Nucl. Eng. Des..

[8] Ronen Y (1989). High Converting Water Reactors.

[9] Carelli, M. *et al*. IRIS, International new generation reactor. In *Proceedings of the 8th* (2023).

[10] Asou M, Porta J (1997). Prospects for poisoning reactor cores of the future. Nucl. Eng. Des..

[11] Yoshida, N., Kawakami, M., Hiraiwa, K. & Heki, H. Study on long life core with uranium fuel for LSBWR. In *Proceedings of PHYSOR*, pp. 1–11, Seoul, Korea, (2002).

[12] Saccheri, J. G., Todreas, N. & Driscoll, M. A tight lattice, epithermal core design for the integral PWR. In *Proceedings of the 2004 International Congress on Advances in Nuclear Power Plants-ICAPP'04* (2004).

[13] Hibi K, Shimada S, Okubo T, Iwamura T, Wada S (2001). Conceptual designing of reduced-moderation water reactor with heavy water coolant. Nucl. Eng. Des..

[14] Yoshida, N., Hiraiwa, K., Nakamaru, M. & Heki, H. Fuel and core design for long operation cycle simplified BWR (LSBWR). In *International Congress on Advanced Nuclear Power Plants (ICAPP), Hollywood, Florida* (2002).

[15] Dandi A, Lee M, Kim MH (2020). Feasibility of combinational burnable poison pins for 24-month cycle PWR reload core. Nucl. Eng. Technol..

[16] Franceschini F, Petrović B (2009). Fuel with advanced burnable absorbers design for the IRIS reactor core: Combined Erbia and IFBA. Ann. Nucl. Energy.

[17] Oettingen M, Cetnar J (2014). Validation of gadolinium burnout using PWR benchmark specification. Nucl. Eng. Des..

[18] Mustafa SS, Amin E (2020). Simulating IRIS assembly containing erbium oxide as a burnable poison. Mater. Today Commun..

[19] Schulz TL (2006). Westinghouse AP1000 advanced passive plant. Nucl. Eng. Design.

[20] Wagner, J. C. & Parks, C. V. Parametric study of the effect of burnable poison rods for PWR burnup credit. In *Division of Systems Analysis and Regulatory Effectiveness* (2002).

[21] Inoue, Y. Combining thorium with burnable poison for reactivity control of a very long cycle BWR. In *Master of Science in Nuclear engineering at the MIT* (2004).

[22] Allen, K., Tulenko, J., Baney, R., Butt, D. & Kim, J. An advanced burnable poison for pressurized water reactors. In *Proceeding Conference, Advances in Nuclear Fuel Management III* 5–8 (2003).

[23] Heki, H. *et al*. Long operation cycle simplified BWR. In *Proceedings of the 9th International Conference on Nuclear Engineering ICONE-9, Nice, France* (2001).

[24] Rao, A., Sawyer, C. & McCandless, R. Simplified boiling water reactor design. In *The 1st JSME/ASME Joint International Conference on Nuclear Engineering* (1991).

[25] Hendricks, J.S. *et al.* MCNPX 2.6. 0 Extensions. Los Alamos National Laboratory, P.73, LA-UR-08-2216 (2008).

[26] Shultis, J. K. & Faw, R. E. An MCNP primer. In *Department of Mechanical and Nuclear Engineering, Kansas State University, Manhattan, KS 66506* (2011).

[27] Rhodes, J., Smith, K. & Lee, D. CASMO-5 development and applications. In *Proceedings of the PHYSOR-2006 Conference, ANS Topical Meeting on Reactor Physics (Vancouver, BC, Canada, 2006) B, Vol. 144* (2006).

[28] Slavickas A, Pabarčius R, Tonkūnas A, Stankūnas G (2014). Decomposition analysis of void reactivity coefficient for innovative and modified BWR assemblies. Sci. Technol. Nucl. Install..

[29] Syarifah, R. D., Suud, Z., Basar, K. & Irwanto, D. Fuel fraction analysis of 500 MWth gas cooled fast reactor with nitride (UN-PuN) fuel without refueling. In *Journal of Physics: Conference Series, Vol. 799* 012022 (IOP Publishing, 2017).

[30] Alam SB (2019). Small modular reactor core design for civil marine propulsion using micro-heterogeneous duplex fuel. Part II: Whole-core analysis. Nucl. Eng. Design.

[31] Peakman A, Owen H, Abram T (2021). Core design and fuel behaviour of a small modular pressurised water reactor using (Th, U) O2 fuel for commercial marine propulsion. Prog. Nucl. Energy.

